# Conjugated Hyperbilirubinemia in a Patient With COVID-19: A Case Report and Literature Review

**DOI:** 10.7759/cureus.96404

**Published:** 2025-11-09

**Authors:** Haider Ghazanfar, Faryal Altaf, Ali Ghazanfar, Bhavna Balar

**Affiliations:** 1 Internal Medicine, BronxCare Health System, Bronx, USA; 2 Internal Medicine, Federal Medical and Dental College, Islamabad, PAK; 3 Gastroenterology, BronxCare Health System, Bronx, USA

**Keywords:** coronavirus disease 2019, gastrointestinal symptoms, hyperbilirubinemia, liver dysfunction, transaminitis

## Abstract

Coronavirus disease 2019 (COVID-19) was first reported at the end of 2019 and soon evolved into a deadly pandemic of the century. It has a myriad of clinical presentations and is associated with significant mortality and morbidity. We report a case of a 49-year-old Hispanic man who presented to the emergency department with complaints of productive cough, shortness of breath, fever, and body aches for the past five days. On clinical examination, he was noted to have jaundice. Laboratory results revealed conjugated hyperbilirubinemia and elevated alkaline phosphatase. The patient tested positive for COVID-19 and appropriate treatment was initiated as per hospital protocols. All potential causes of hyperbilirubinemia were systematically excluded, including autoimmune, metabolic, hemolytic, septic, drug-induced, parasitic, and biliary etiologies. Hyperbilirubinemia improved with the treatment of COVID-19. All other causes of hyperbilirubinemia must be excluded in these individuals before attributing it to COVID-19. Further research is needed to understand the disease and to guide appropriate therapy.

## Introduction

Coronavirus disease 2019 (COVID-19) first emerged in China at the end of 2019 and soon became a major global health crisis. It has been associated with significant mortality and morbidity [1]. COVID-19 infection has a wide spectrum of clinical presentations ranging from asymptomatic carriers to gastroenteritis, severe pneumonia, pulmonary embolism, stroke, multi-organ failure, sepsis, septic shock, and death [2,3].

A significant number of patients with COVID-19 have presented with gastrointestinal symptoms. A multi-center cross-sectional study concluded that almost 50.5% of the patients reported digestive symptoms [4]. Lack of appetite and diarrhea are the most common gastrointestinal manifestations [4,5]. Nausea, vomiting, abdominal pain, and gastrointestinal bleeding are the least reported clinical symptoms of COVID-19 [4,6]. We present a case of a 49-year-old man with COVID-19 and hyperbilirubinemia.

## Case presentation

A 49-year-old man presented to our emergency department with complaints of productive cough, shortness of breath, fever, and body aches for the past two days. His symptoms have progressively worsened with frequent fever spikes and myalgias. He reported associated nausea and vomiting for the last two days. He also developed non-bloody watery diarrhea for the past day. The patient's past medical history was significant for human immunodeficiency virus (HIV) with a CD4 count of greater than 300, chronic migraines, bipolar disorder, and carpal tunnel syndrome. The patient had no changes in medications over the past year. The patient’s surgical history included an appendectomy. He denied any significant family history. The patient denied any smoking, alcohol use, or recreational substance use. He had no sick contact or any recent travel history. In the Emergency Department, the patient had a temperature of 103°F, a heart rate of 125 beats per minute, blood pressure of 124/84 mmHg, a respiratory rate of 18 breaths per minute, and oxygen saturation of 92% on room air. On general physical examination, the patient was found to be icteric. Respiratory examination was significant for bilateral basal crackles. The patient’s abdominal examination was unremarkable. Patients’ initial laboratory findings are presented in Table 1. Patient's baseline labs were within normal limits.

**Table 1:** Initial Laboratory Findings

Complete Blood Count	Value
White Blood Cell Count	12.4 k/ul (4.8 - 10.8 k/ul)
Hemoglobin	10.1 g/dl (12.0 - 16.0 g/dl)
Hematocrit	30.8% (42.0 - 51.0%)
MCV	84.1 fL (80.0 - 96.0 fL)
MCH	27.5 pg (27.0 - 33.0 pg)
MCHC	32.8 g/dl (33.0 - 36.0 g/dL)
Platelets	382 k/ul (150 - 440 k/uI)
Electrolytes	
Sodium	136 mEq/L (135 - 145 mEq/L)
Potassium	4.1 mEq/L (3.5 - 50 mEq/L)
Bicarbonate	20 mEq/L (24 - 30 mEq/L)
Chloride	97 mEq/L (98 - 108 mEq/L)
Glucose	133 mg/dL (70 - 120 mg/dL)
BUN	68 mg/dL (8 - 26 mg/dL)
Creatinine	3.0 mg/dL (0.5 - 1.5 mg/dL)
Thyroid-Stimulating Hormone	2.41 (0.40 – 4.50 mIU/L)
Liver Function Test	
Albumin	2.7 g/dl (3.4 - 4.8 g/dlL)
Bilirubin, Total	14.7 mg/dL (0.2 - 1.2 mg/dL)
Bilirubin, Conjugated	11.9 mg/dL (0.0 - 0.3 mg/dL)
Alkaline Phosphatase	476 unit/L (56 - 119 unit/L)
Gamma Glutamyl Transferase	78 unit/L (8 - 54 unit/L)
Aspartate Transaminase	391 unit/L (9 - 48 unit/L)
Alanine Aminotransferase	101 unit/L (5 - 40 unit/L)
Total Protein	8.1 g/dl (6.0 - 8.5 g/dl)

Ultrasound of abdomen with Doppler showed fatty infiltration of the liver and patent main portal vein with normal direction of the blood flow. CT abdomen and pelvis without contrast showed hepatomegaly and patchy infiltration in the lower right lobe of the lung. The patient tested positive for COVID-19 and was started on the COVID-19 treatment protocol, which included broad-spectrum antibiotics including vancomycin and piperacillin-tazobactam, dexamethasone, Remdesivir, and supplemental oxygen. Abdominal MRCP showed no evidence of cholelithiasis, choledocholithiasis, or biliary ductal dilation. Blood, urine, and respiratory culture showed no growth of any organisms. The patient tested negative for IgM Hepatitis A and B, Hepatitis B surface antigen and Hepatitis B core antibody. In fact, the patient was found to have immunity to Hepatitis B. He had negative Hepatitis C antibody as well. His peripheral smear was unremarkable. He also had a normal haptoglobin and reticulocyte count. Autoimmune and metabolic work-up was negative. This is presented in Table 2.

**Table 2:** Autoimmune and Metabolic Work-up

Investigation	Value
Anti-nuclear Antibodies	Negative
Anti-mitochondrial Antibody	Negative
Smooth Muscle Antibody	Negative
Immunoglobulin G	821 (600 - 1640 mg/dL)
Immunoglobulin A	224 (47 – 310 mg/dL)
Ceruloplasmin	33 (18 - 36 mg/dL)
Liver Kidney Microsomal Assay	< = 20.0 (< = 20.0)
Celiac Panel	No Serological Evidence for Celiac Disease Is Present

The patient’s liver function tests began to improve in parallel with the overall clinical recovery. The patient remained hospitalized for a total of seven days and was subsequently reviewed in the outpatient clinic one week after discharge. Laboratory investigations were obtained on the day of discharge (Day 7) and repeated as an outpatient on Day 10. The trends in COVID-19 inflammatory markers and liver function tests are summarized in Table 3.

**Table 3:** COVID-19 Inflammatory Markers and Liver Function Tests COVID-19: Coronavirus disease 2019.

Investigation	Day 1	Day 3	Day 7	Day 10	Normal Values
COVID-19 Inflammatory Markers					
C-Reactive Protein	>350.0			10.40	< = 5.00 mg/L
Ferritin	4297.0			2279.0	100 - 190 unit/L
Lactate Dehydrogenase	583			259	13.0 - 150.0 ng/mL
D-Dimer	2079			355	0 - 230 ng/mL
Liver Function Tests					
Bilirubin, Total	14.7	11.7	3.1	1.2	0.2 - 1.2 mg/dL
Bilirubin, Conjugated	11.9	10.0	2.2	0.8	0.0 - 0.3 mg/dL
Alkaline Phosphatase	476	348	265	104	56 - 119 unit/L
Aspartate Transaminase	391	143	64	42	9 - 48 unit/L
Alanine Aminotransferase	101	101	103	61	5 - 40 unit/L

**Figure 1:**
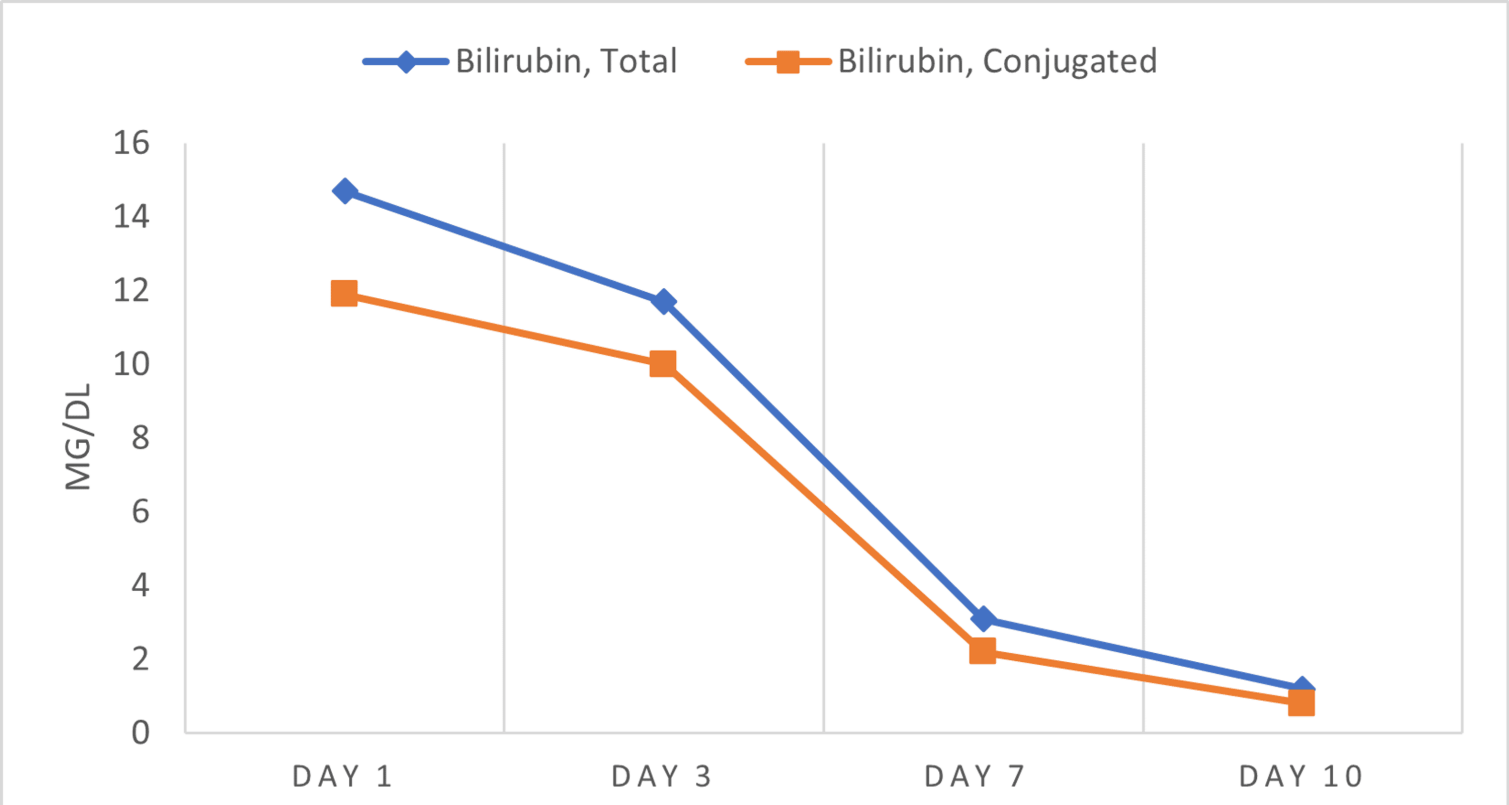
Trend of Total and Conjugated Bilirubin

**Figure 2:**
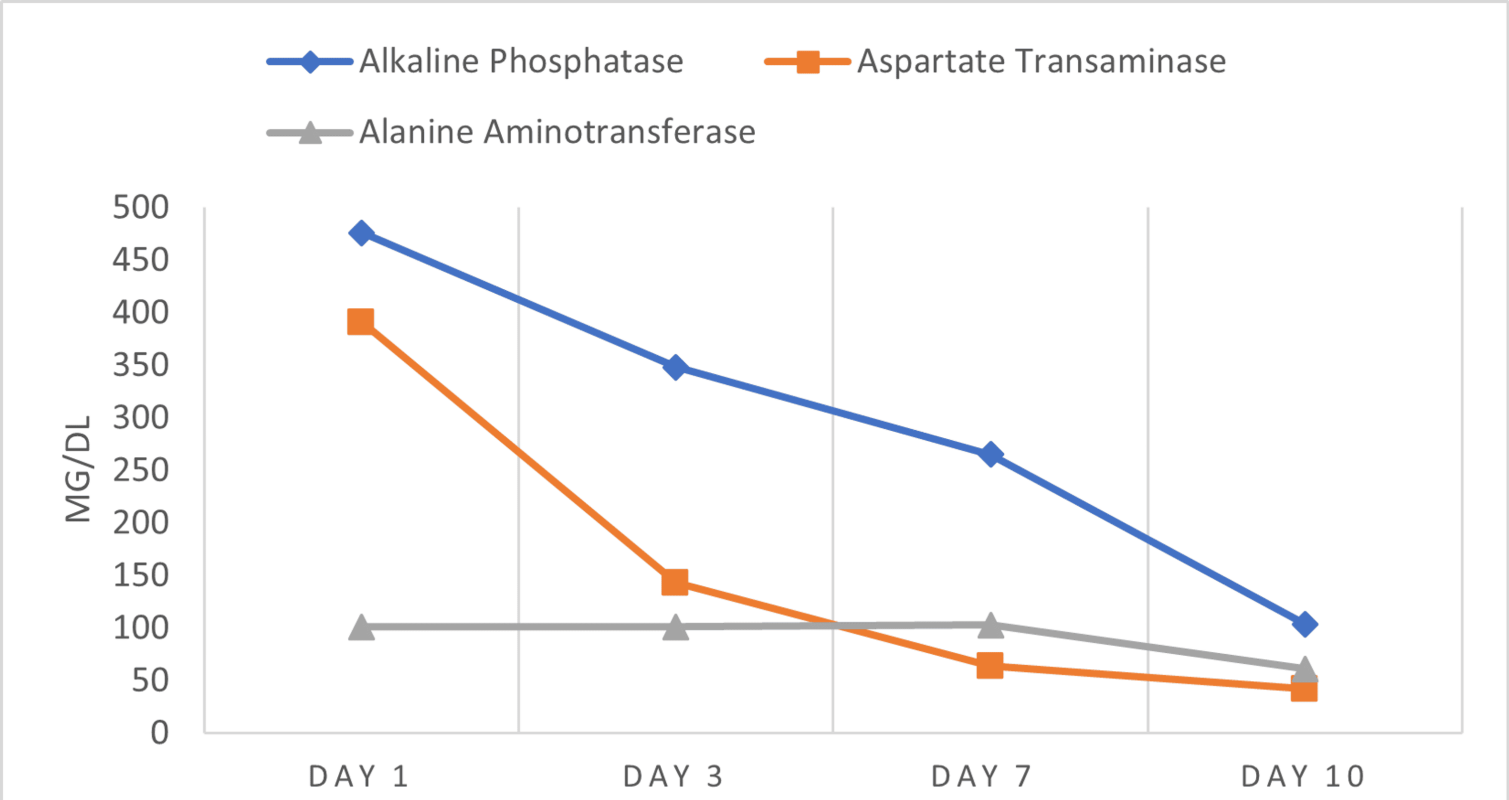
Trend of Alanine Aminotransferase, Aspartate Transaminase, and Alkaline Phosphatase

The trend of the liver function test is presented in Figures 1, 2.

## Discussion

COVID-19 infection has been shown to affect multiple organ systems including the hepatobiliary system. Studies have shown that approximately 60% of patients with COVID-19 infection experience liver damage, significant by elevated liver enzymes [7]. A study done in China showed that severe acute respiratory syndrome coronavirus 2 (SARS-CoV-2) particles can be seen inside the hepatocytes, confirmed by using probes specific for a SARS-CoV RNA polymerase gene fragment [8]. The study proposed that one of the mechanisms underlying these pathological changes in the affected organs may be a direct cytopathic effect mediated by the local replication of the SARS-CoV-2 virus. Patients with severe COVID-19 have been shown to have a higher rate of liver dysfunction. According to a study done by Huang et al., it was concluded that 62% of the patients admitted to the intensive care unit (ICU) had elevated aspartate aminotransferase as compared to 25% of patients who did not require ICU-level care [9]. According to a pooled analysis, it was shown that patients with severe COVID-19 had a higher bilirubin concentration as compared to patients without severe disease (p = 0.001) [10]. Studies have been done to assess the association of hyperbilirubinemia with COVID-19 severity. Results of some of the studies have been presented in Table 4 [2,11-13].

**Table 4:** Review of Studies Demonstrating Correlation of Hyperbilirubinemia With the Severity of COVID-19 COVID-19: Coronavirus disease of 2019

Study	Study Type	Sample Size	Cut-off Point Defining Abnormal Bilirubin Value	Severe Patients with Abnormal Bilirubin Values	Non-severe Patients with Abnormal Bilirubin Values	p-Value
Guan et al. [2]	Retrospective Study	722	>17.1 mmol/L	13%	99.30%	NA
Cai et al. [11]	Cross-sectional Study	318	>17.1 mmol/L	75.30%	60.10%	< 0.001
Li et al. [12]	Ambispective Cohort Study	541	>21 mmol/L	6.40%	2.50%	0.036
Zheng et al. [13]	Retrospective Study	161	>20.5 mmol/L	10.00%	4.60%	0.244

Various mechanisms have been suggested for liver dysfunction associated with COVID-19. SARS-CoV-2 can either cause direct hepatotoxicity or can indirectly cause liver dysfunction following septic shock, hypoxic injury, multi-organ dysfunction, microthrombosis of hepatic sinusoids, systemic inflammatory response or secondary to drug-related toxicity [14]. According to a study, the liver biopsy of the COVID-19 patients showed moderate micro-vesicular steatosis [15].

In a retrospective study done on 79 COVID-19 patients, it was shown that male patients were more likely to develop liver injury (p < 0.05) [16]. In the same study, it was concluded that the patient with liver damage had a longer length of hospital stay as compared to the patient who did not have the liver disease (15.4 vs 11.4) (p < 0.05) [16]. Another study had a similar finding (p = 0.021) [17].

According to the review of literature to date, it has been concluded that the treatment of patients with liver damage should be targeted towards COVID-19. Symptomatic support, rational oxygen therapy, antiviral therapy, and anti-infective agents have been advised for these patients [18]. It is important to identify the underlying cause of liver injury in these patients so that appropriate treatment can be promptly started. Glycyrrhizic acid has been shown to have antiviral activity against SARS-CoV-2 [19]. Studies have shown promising results for using glycyrrhizic acid in patients with liver disease because of the drug’s anti-inflammatory and hepato-protective effects [19,20]. In this case, the patient’s liver dysfunction showed gradual improvement following appropriate management of the underlying COVID-19 infection.

It has been reported that COVID-19 patients with liver dysfunction have higher mortality and morbidity [21,22]. It is important to closely monitor these patients for the development of acute liver failure. Patients who presented with gastrointestinal symptoms of diarrhea, nausea, and vomiting were more likely to develop severe COVID-19 [21]. 

## Conclusions

COVID-19 has been increasingly recognized as a multisystem disease with potential hepatic involvement. Our case underscores the importance of early recognition and prompt management of COVID-19-related liver dysfunction. Equally important is the exclusion of alternative etiologies of transaminitis to ensure accurate diagnosis and appropriate treatment. Patients with COVID-19-associated liver injury have been reported to have more severe clinical courses, highlighting the need for vigilant monitoring and timely intervention. There is a need for further mechanistic and prognostic studies to elucidate the association between elevated bilirubin levels and the severity of COVID-19.
